# Electron Transfer and Negative Ion Formation

**DOI:** 10.1021/acs.jpclett.5c01686

**Published:** 2025-07-24

**Authors:** Paulo Limão-Vieira, Gustavo García

**Affiliations:** † Atomic and Molecular Collisions Laboratory, CEFITEC, Department of Physics, 50106 Universidade NOVA de Lisboa, 2829-516 Caparica, Portugal; ‡ Instituto de Física Fundamental, 16379 Consejo, Superior de Investigaciones Científicas (CSIC), Serrano 113-bis, 28006 Madrid, Spain

## Abstract

The role of electron transfer (ET) is crucial in determining the fate of chemical reactions within a diversity of scientific domains, encompassing fundamental and applied research. In such processes where anion formation prevails, the rather complex mechanisms occurring at the molecular level still pose challenges to both experimentalists and theoreticians. Here, we address negative ion formation from crossed molecular beam experiments in a wide collision energy range, from a threshold up to a few keV, noting important aspects of the collision dynamics. This Perspective is not intended to give a review on electron transfer and negative ion formation but the authors’ major contributions in atom–molecule and anion–molecule collision experiments, while also highlighting the most relevant achievements and challenges in some dedicated research fields that are still impacting many researchers across the globe.

## Graphical abstract

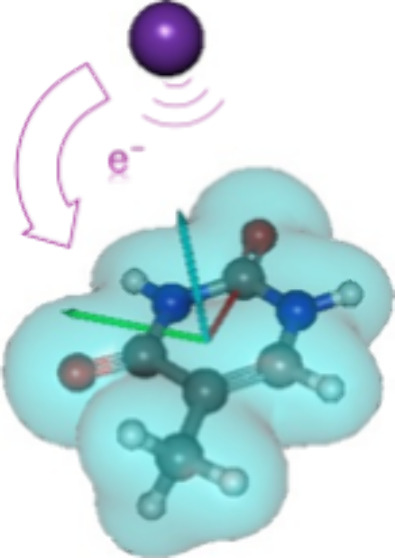

Electron-transfer (ET) processes have been widely recognized as prevalent in different environments as a function of phase and stage of aggregation.^1^ These are responsible for triggering chemical reactions either on natural (physical, biological, environmental) or industrial processes in a wide variety of media, including, e.g., the formation of organic molecules within ice mantles on dusty grains in the interstellar medium,^2,3^ the control of fluorocarbon plasmas used to produce silicon chips,^4^ graphene related nanofunctionalized electrode materials,^5^ scanning tunnel microscopy,^6^ radiation science and medicine,^7–9^ photochemistry of adsorbed molecules,^10,11^ molecular electronics for photovoltaics,^12^ and energy storage.^13^ ET (and even charge transfer) processes to atoms and molecules can be probed in many different environments while preparing the collision partners under energy state selective conditions. As an example, in the gas phase, such interactions can be investigated in collisional interactions between neutral atom and neutral molecule and between anion and neutral molecule, low-energy reactions of charged particles, ion traps, electrospray conditions, photofragmentation and photodetachment,^14^ among many others. Also relevant in the past decade are aspects of electron-transfer-mediated decay (ETMD) which have been shown to be important in, e.g., helium nanodroplets,^15,16^ liquid microjets,^17^ and the condensed phase^18^ (see also references therein). ETMD usually occurs after photoionization of heterogeneous nanosystems; here, an electron from a neighboring species will occupy a core hole. This has implications as to the detailed knowledge of radiation damage in biomolecular systems, where extensive local water ionization ETMD of solvated ions may initiate a cascade of highly reactive radical reactions.^17^ Notwithstanding, ET in the gas phase employing molecular beams femtochemistry, have allowed us to unveil the rather fascinating atomic scale dynamics of chemical bonds.^19^ Also, and within the relevance of the electronic structure of molecules, important and additional methodologies have been employed. Rydberg electron-transfer (RET) processes coupled with state-of-the art velocity map imaging (VMI) techniques have been pivotal for obtaining electron binding energies from anion photoelectron spectra, with significance to dipole-bound^20–22^ and more recently to quadrupole-bound anion states.^23^ Currently, the new uprising of attochemistry on the ultrafast electron dynamics will be essential to broaden our knowledge on the natural time scale of electronic motion in matter, and as recently reported, on the role of electron hydration in charge-transfer-to-solvent states in aqueous conditions,^24^ with important consequences for controlling chemical reactions.

The role of collisional processes within plasma physics, astrophysics, environmental and biological sciences has been decisive to shed light on the underlying molecular mechanisms where photons and electrons prevail to initiate reactions.^4,7^ Anion–molecule interactions represent a different collision dynamics mechanism from both free and (weakly) bound electron attachment, which are also responsible for electron-transfer processes. With the major goal to probe key selected molecular targets relevant within the scope of astrochemistry and even biological environment constituents (e.g., ethanol, methanol, water, molecular oxygen, carbon dioxide, benzene), authors have been using different projectiles as H^–^, O^–^, HO^–^ and more recently the superoxide anion O_2_^–^ to obtain branching ratios of positive and negative ions formed in such collisional processes^25–29^ (and references therein). A schematic representation of the Madrid experimental set up is shown in Figure 1. Interesting to note are also absolute electron detachment, relative total and partial ionization cross sections being obtained for O_2_, CO_2_ and C_6_H_6_.^26,28,29^ In the case of carbon dioxide and benzene, an energy dependent double ionization of the target molecule followed by electrostatic attraction with the anionic projectile yielding charged complexes with *m*/*z* ratios greater than the parent molecules, have been reported.^26,27^ Nonetheless, for the rather different collision energy ranges (typically above a few hundreds of eV up to several keV) from ion-pair formation experiments (from threshold up to 0.5 keV), no further details or discussion will be given.

**Figure 1:**
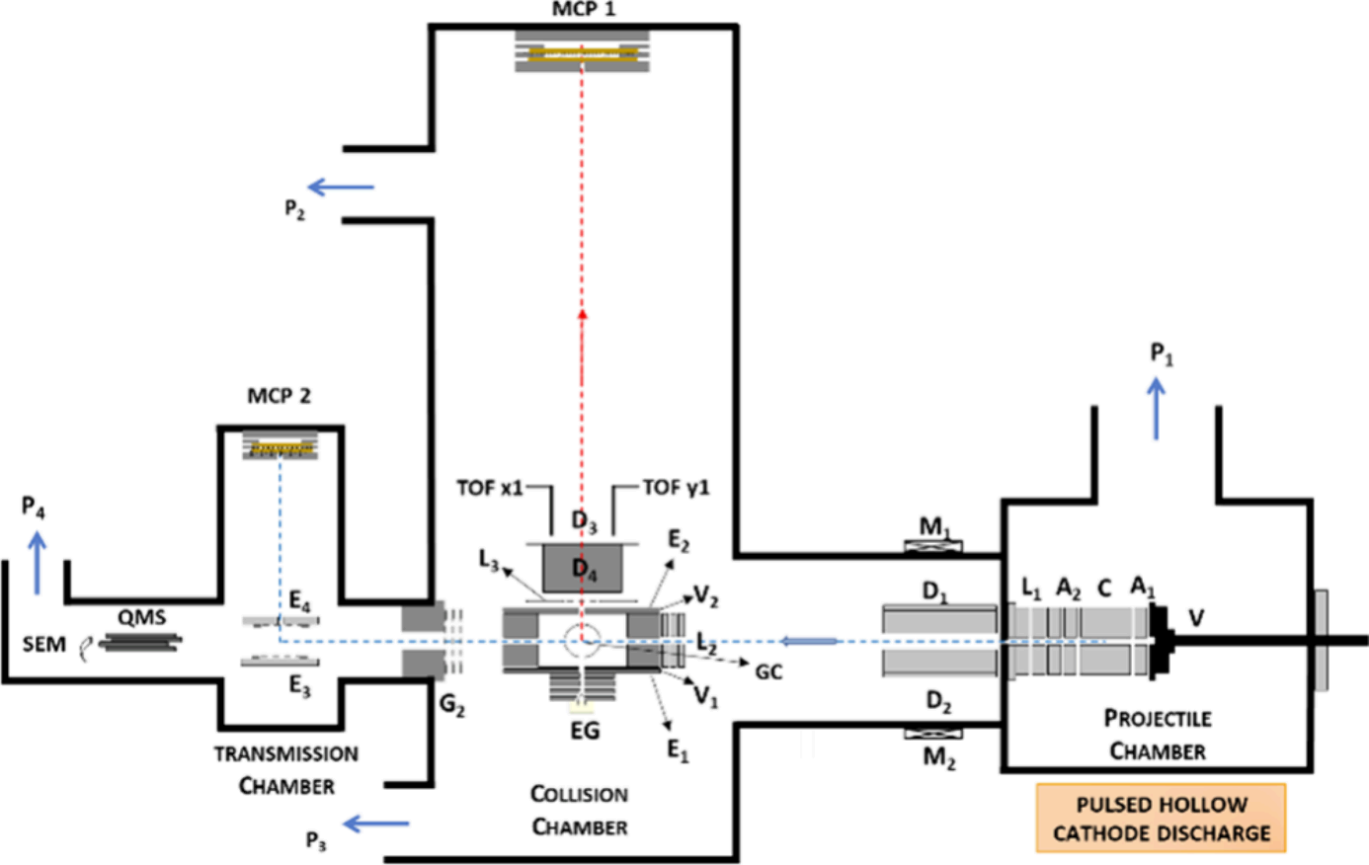
Schematics of the Madrid experimental setup for anion-neutral molecule collision experiments. V – pulsed valve; C – hollow cathode discharge source; A_1_, A_2_ – anodes; L_1_, L_2_, L_3_ – Einzel lenses; D_1_, D_2_, D_3_, D_4_ – deflecting plates; M_1_, M_2_ – magnets; E_1_, E_2_, E_3_, E_4_ – extraction plates; G2 – focusing grids; EG – electron gun; GC – gas-cell; MCP 1, MCP 2 – multichannel plate detectors; QMS – quadrupole mass spectrometer; SEM – secondary electron multiplier detector; P_1_, P_2_, P_3_, P_4_ – turbomolecular pumping system.

During the last decades, the most relevant advent of electron induced processes has been related to DNA genotoxic damage by subionizing low-energy electrons (LEEs),^30,31^ and the multitude of different scientific outputs (experiment and theory) related to biological molecules in the gas, cluster and condensed phases, which have been reported across the globe. For a thorough description see Baccarelli et al.,^32^ Alizadeh et al.,^33^ Kumar and Sevilla,^34^ Arumainayagam et al.,^35^ and references therein. However, the relative intricate underlying molecular mechanism that may lead to loss of DNA integrity, at least for electron energies below 15 eV has been recognized to be solely due to an electron-transfer process from a shape or core-excited resonance on a nucleobase to the sugar phosphate group, yielding C–O bond excision via a dissociative electron attachment process.^33^ Moreover, in such an electron energy range, local electronic excitation can also give rise to dissociation, where a single low-energy electron can break two bonds.^33^ Pulse radiolysis has shown the nonrelevant effect of solvated electrons in biological damage; however, the role of prehydrated electrons that can promptly be transferred to solute molecules and even lead to DNA damage, with and without radiosensitizers, in hydrated conditions still needs to be properly assessed.^33,36,37^ We have been noticing a rapid increase in the investigations related to the underlying molecular mechanisms responsible for radiosensitization, an important process within chemoradiation therapy. Additionally, drug design and tailor-made protocols still lack from detailed information about the prevalence of low-energy electrons (and presolvated electrons) and the role of electron-transfer processes to specific sites of DNA in cancer cells.^38^

Neutral atom–neutral molecule collisions may yield reactive, ion-pair formation, and even other inelastic scattering processes, which are certainly energy dependent. ET processes resulting in ion-pair formation requires a threshold energy Δ*E* which is given by the difference of the ionization energy of the electron donor atom and the electron affinity of the acceptor target molecule.^39^ We shall not describe the background of electron transfer yielding ion-pair formation given so many references are available in the literature but rather suggest key selected contributions as references 39–42 and those cited therein.

In the last 20 years, we have thoroughly investigated negative ion formation in electron-transfer experiments involving neutral potassium atoms and neutral polyatomic molecular targets (Figure 2). The comprehensive nature of the experimental studies together with the aid of quantum chemical calculations have allowed investigation of the underlying molecular mechanisms yielding fragmentation of selected molecular targets with major relevance, but not only, to biomolecules. Such achievements include novel electron-transfer induced fragmentation patterns of thymine and uracil,^43^ with the most striking difference from previous dissociative electron attachment (DEA) results being the enhanced yield of anions stemming from bond breaks in the pyrimidine ring. Of relevance, in terms of controlling and inducing selectivity of chemical reactions in molecular collisions, it was shown that, at room temperature and with random molecular orientation, site (N1–H vs N3–H) and bond (C–H vs C–N) selective dissociation in DNA/RNA pyrimidine nucleobases can be achieved by tuning the proper collision energy.^44^ Such a site and bond selectivity process is not only restricted to biological molecules as the DNA base adenine,^45^ while operative in other target molecules as, e.g., the anesthetic halothane^46^ and some nitroimidazolic radiosensitizers.^47,48^

**Figure 2:**
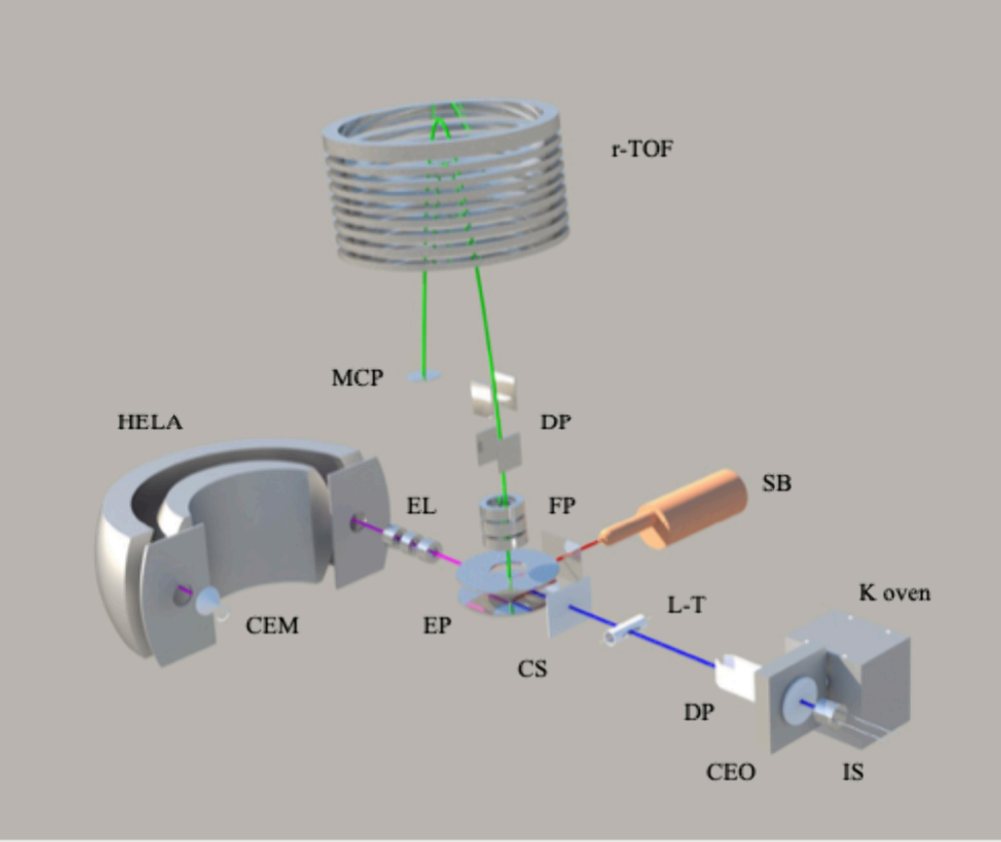
Schematics of the Lisbon experimental setup for neutral atom-neutral molecule collision experiments. IS – K^+^ ion source; K oven – potassium oven; CEO – charge exchange oven; DP – deflecting plates; L-T – Langmuir–Taylor detector; CS – collimating slits; EP – extraction plates; SB – secondary beam source; FP – focusing plates; EL – Einzel lenses; CEM – channel electron multiplier; HELA – hemispherical energy loss analyzer; MCP – multichannel plate detector; r-TOF – reflectron time-of-flight.

Later joint ET and DEA experiments on 1- and 3-methylthymine and 1- and 3-methyluracil, together with *ab initio* calculations have shown the dynamics of the decaying transient anion and the relevant competition between dissociation and autodetachment.^49^ Within the context of ET, the collision energy dependent N3–CH_3_ bond excision was shown to be a predominant mechanism of an initial electron capture to the π* state and subsequent intramolecular transfer to a σ* state. Moreover, NCO^–^ branching ratios as a function of the collision energy are reminiscent of extraordinary site- and bond-selectivity in the reactions yielding its formation.^50^ These findings allowed us to establish a new collision induced dissociation mechanism for DNA damage that may be described at a basic molecular level. This model also provides a coherent explanation of the observed correlation between electron transfer to biomolecules and their carcinogenicity and may be used to suggest new compounds to be adopted in radiation therapy as treatment enhancing sensitizers. Radiosensitization properties of halouracils (e.g., 5-XU, X = F, Cl, Br, I) have been known for several decades with irradiation of cells in which some DNA thymines have been replaced by halogenated uracils, increasing the frequency of both single and double strand breaks.^51^ The electron-transfer model provides an explanation for such effects with the introduction of a strongly electrophilic atom into the DNA (e.g., F, Cl), leading to an enhancement in the collision induced dissociation probability and thence an increased probability for DNA destruction in cells containing such compounds.^52^ Another relevant aspect of the underlying radiosensitization mechanisms which are not known in detail yet pertains to the role of selected nitroimidazolic chemical compounds more attuned to hypoxic cells, e.g., nitroimidazoles^47,53^ and nimorazole.^38,48,54^ These upon electron transfer exhibit parent anion formation strongly competing with dissociation and even suppressing fragmentation upon hydration.^38^ Notwithstanding, the role of these chemical compounds which may be incorporated in cells prior to irradiation of the biological material requires a proton transfer to the reduced chemical compound, yielding a neutral radical species that will bind to DNA, resulting therefore in strand breaks.^38^

In order to obtain the fragmentation patterns in the DNA/RNA sugar unit analogue and assess the major significance of the decomposition mechanisms, our other contributions include investigations into tetrahydrofuran (THF)^55^ and d-ribose.^56^ Here we have observed ring breaking as the main decomposition channel, in contrast to results from DEA experiments.^57^ Special emphasis was also given to the dissociation mechanisms lending support to the breaking of the N-glycosidic bond, as an initial step in the fragmentation of the temporary negative ion (TNI) in uridine.^58^ These studies have shown that electron-transfer processes are much more effective than DEA in producing a loss of integrity within DNA/RNA units. As far as the small amino acids are concerned, neutral OH loss in glycine was observed^59^ whereas in small aliphatic amino acids (alanine and valine), the differences observed are due to the higher number of degrees of freedom of the side chain. In the case of valine, that can be linked with the formation of lighter fragments when the fragmentation process proceeds through a statistical dissociation.^60^ In DEA studies to several (bio)­molecular targets, the dominant fragmentation channels result from very low-energy resonances (often as low as ∼0 eV) consisting of vibrational Feshbach resonances.^61^ This can be rationalized by the fact that, in DEA, accessing high-energy resonances (such as NCO^–^ formation in uracil/thymine^43^) will mostly result in autodetachment, rather than in fragmentation; such is also the case for, e.g., d-ribose.^56^ However, in electron-transfer from potassium (K)–molecule (ABC) collisions, there is evidence that the TNI autodetachment may be significantly suppressed due to the Coulomb interaction in the collision complex (K^+^ABC^–^) enhancing fragment formation. Such a process is certainly collision energy dependent, being more favorable at lower rather than higher energies.

The collision time is set between the alkali approach upon electron transfer and departure from the target, and this may also be of the order of vibrational periods within the target molecule coordinates. In such relevant vibronic coupling,^40^ yielding bond excision, the collision process may be dictated by a rather statistical than direct dissociation mechanism. In ion-pair formation, the reaction thermodynamic thresholds can result in energy values lower than the experimental evidence. Thus, momentum conservation of the dissociating partners may impact the lighter kinetic energy, accounting for such differences. Within the TNI unimolecular decomposition, kinetic energy release distributions (KERDs) for H^–^ from methanol,^62^ ethanol,^63^ water,^64^ F^−^ and Cl^−^ from halogenated benzenes and derivatives,^65,66^ and O^−^ and OH^−^ from tetrahydrofuran and d-ribose,^67^ were reported. The comprehensive analyses revealed the role of statistical and direct dissociation in the collision process, where the excess energy was either converted into the available degrees of freedom with average values of ≅0.5 eV or being channelled into translational energy of fragments formed with an excess of >1.5 eV.

Another relevant aspect relates to the rate of chemical reactions which are dependent on the importance of transition states^68–70^ (and references therein). In electron-transfer processes yielding ion-pair formation, the ion-pair/polarization-interaction in the collision complex may not only influence the lifetime of the TNI formed but may also stabilize specific transition states and thus noticeably modify the fragmentation patterns. We have noticed that electron transfer to acetic acid yielding OH^–^, requires considerable internal rearrangement within the TNI, which is initially triggered by hydrogen scrambling from the methyl group to the carboxylic group.^71^ The experimental evidence combined with post-Hartree–Fock and Density Functional Theory (DFT) calculations have shown that hydroxyl anion formation proceeds through the most favorable intermediate mechanism via diol formation.

As far as electron-transfer induced side chain cleavage in tryptophan is concerned, within the unimolecular decomposition of the TNI, the major dissociation channel was assigned to dehydrogenated parent anion formation.^72^ The role of the collision complex in the electron-transfer process is significant for mechanisms that operate at lower collision energies, typically below 50 eV. At those collision times, on the order of a few tens of fs, the collision complex may not only influence the lifetime of the TNI but also stabilize specific transition states and thus modify the fragmentation patterns significantly. It is worth noting that the TNI stabilization in the complex is possible; however, a preferable geometry in the collision complex may allow for the required hydrogen-transfer processes to proceed within its lifetime. Therefore, such collision dynamics has important consequences for the dissociation channels attained. These are competitive channels, and as the transfer energy is increased (by increasing the collision energy), an enhancement of direct dissociation processes may occur. This may imply that rearrangement reactions such as the dehydrogenated indoline anion formation can be critically dependent on reaching a favorable geometry, thus allowing for the respective rearrangement to proceed.

Recently, the collision dynamics in K–H_2_O and K–D_2_O have revealed novel important differences in the fragment anions formed, rendering a relevant isotope effect for D_2_O; the character of singly excited molecular orbitals and doubly excited states has also been reported, the latter suggested to be closely related to neutral dissociation.^64^ Such processes seem to be operative in methanol (CH_3_OH),^62^ ethanol (CH_3_CH_2_OH)^63^ and sulfur hexafluoride (SF_6_),^73^ yet further complementary experiments and theoretical calculations on the potential energy surfaces and resonance widths of such states are needed.

Currently we still miss relevant information on the intricate underlying molecular mechanisms triggered by ET, while at the experimental level relevant emphasis has been put on the fragmentation yields of ionic species, the signature of resonances, and momentum conservation aspects, among several others. From the theoretical point of view important characteristics of potential energy curves for simple diatomic and even potential energy surfaces for triatomic molecules have been made possible; however, the (prohibitive) computational cost is still limiting the capacity to perform more sophisticated calculations. However, more robust and even different calculation strategies will need to include many body systems, such as electron–electron and spin–orbit couplings, to help unravel the intricate landscape of electronic and nuclear motion in such polyatomic molecular targets upon electron transfer.

With this Perspective we hope to trigger both the interest and discussion of the following aspects:

- Dissociative electron transfer (DET) in atom–molecule collisions experiments has been shown in several occasions;^44^ however, such a process is not restricted to the gas phase and may have an important role within doped solids and liquids, and the surface of dielectric solid films, within the few fs time scale.^33^
- Within the atom–molecule collisions realm, nonadiabatic processes have been widely recognized as central in electronic and vibrational energy transfer. Several efforts have been made in semiclassical analytic models dealing with atom–atom and atom–molecule collisions being recently revised and improved,^74,75^ while being complementary to quantum scattering calculations. Moreover, from these models, cross sections and rate coefficients of electronic excitation and quenching for collisional energy transfer can be obtained in strongly nonequilibrium environments (e.g., hypersonic shock waves and low-temperature plasma flow reactors); however, they still require validation from the available reliable *ab initio* potentials and the experimental data.^74^
- Other relevant processes are related to vibrational energy transfer which are ubiquitous in, e.g., gas discharges, molecular lasers, plasma chemical reactors and physics of the upper atmosphere.^75^ Adamovitch et al.^75^ have noted relevant developments to kinetic modeling calculations for NO, O_2_ and CO, stressing the need for further experimental data to validate and even benchmark the rates of vibration–translation, vibration–vibration–translation energy transfer and highly vibrationally excited states of such molecules.
- Gelfand and co-workers^68^ noted that above the lowest-lying electronically excited states the (still) difficult, and even almost impossible, task to computationally obtain information about transition states. This requires a high level of quantum chemical calculations to obtain information on energies, barriers and local minima as a function of a molecular configuration. Additionally, at those energies, potential energy curves along dedicated coordinates are no longer harmonic, thus yielding strong vibronic coupling and so more complex dynamics.^68^ This is computationally intractable even for diatomics and triatomics.^69^
- For ET processes yielding ion-pair formation, the current state-of-the art quantum chemical calculations still do not reflect the experimental observations as to the formation and evolution of the collision complex determining the transition states and the reaction paths. This is a dynamic problem, in which the theoretical description may require molecular dynamics simulations or biased-sampling techniques.^72^ These approaches are computationally expensive but could initially be performed on more simple systems rather than on the polyatomics noted above and, thus, could be benchmarked with the experimental data.
- Although we know reasonably well electron induced processes (electron attachment) yielding negative ions, not much is known about neutral dissociation cross sections. These are certainly prevalent in different environments as in electron induced reactions in, e.g., Focused Electron Beam Induced Deposition (FEBID) processes where electronically excited precursors yielding neutral fragmentation may play a crucial role.^76^
- The calculations that are usually performed provide information on electronically excited states, accounting only for a single occupied molecular orbital (MO) being replaced by a nonoccupied (virtual) MO.^48,62–64,73^ Therefore, the role of doubly excited states in ET processes has not been considered yet. Calculations on the potential energy surfaces and resonance widths of such superexcited states would be extremely valuable. This information would also allow us to assess the role of the strong competition between superexcited states and bond breaking into neutral fragments.^77^
- Further experiments, and even theory on collisional electron transfer to clusters, coupled with VMI techniques, will open up a relevant wealth of information bridging the gap between gas and condensed phases;^78,79^ important details of the intermolecular and intramolecular processes within clusters triggered by ET are not known and may be of valuable help to investigate some of the exoplanets atmospheres and even mechanisms within the interstellar medium (ISM).
- The current possibility of making liquid microjets under vacuum conditions appears fascinating from the prospect of exploring new mechanisms within these beams, see, e.g., refs 80 and 81 and references therein. However, the role of a hydrated-like environment that may give a more reliable description of solvent-to-solute ET processes, is still in its early days. The major challenges have been reported to be related to operational conditions in photoelectron spectroscopy, in particular for electron energy spectrometers/analyzers, since the presence of water may compromise the surface potentials also changing the metal surface vapor coverage in these devices.^80^ Moreover, liquids of high viscosity may also impose limitations given the experimental need to have higher pressures prior to nozzle expansion to attain a reasonable flow rate downstream.^81^
- Negative ion collisions with neutrals where not only the relevance within the ISM has been noted^3,82^ but also the importance of charge transfer in collisions in the atmospheres of the many moons and planets that contain atmospheres.^82^ However, there is still a lack of data of anion–neutral molecule collisions with other targets than those reported here. These will be important to investigate the rather complex intermolecular mechanisms which may yield some preferential “quasi-molecular” compounds with *m*/*z* higher than the parent molecule.

With this contribution we hope to have provided a general overview of key selected cases for ET and negative ion formation;^43–49,53,55,56,58,65,67^ however, the examples put forward are by no means a comprehensive description of all activities and achievements that have taken place within the international scientific community. Notwithstanding, the different aspects covered highlight important intramolecular, and even intermolecular, mechanisms where the collision dynamics and the electron-transfer process still require different and more complex approaches at both experimental and theoretical levels.^62–64,73^

The current aspects related to the chemistry (and even physics) of the ISM, the search for forms of *life* and the composition and reaction processes occurring in the atmosphere of exoplanets, appear very exciting and challenging for physicists, chemists, and astronomers. Electron-transfer processes are not yet completely understood, and the increase of space missions to explore these environments, coupled with modern and state-of-the art detection and spectroscopic technologies, opens up the prospect of decades ahead devoted to such investigations.

Within the particular case of radiosensitization, we are at an early stage of describing the underlying molecular mechanisms governing these intricate processes under hydrated and even under cellular conditions.^33,34,38,54,83–86^ The rapid growth of experimental techniques that convey hydration conditions to molecular/cluster beams therefore poses significant challenges to experimentalists but perhaps more to theoreticians. The latter will have to redefine the *traditional* available tools to new calculation approaches, as the computational methodologies demand distinctive installed computing facilities and faster speeds to adapt to this new molecular landscape.^87–89^

Finally, we anticipate that ET will continue to be an important research area of science and technology in the years to come, still impacting the current investigations across the globe but more importantly with the prospect of a wider application as a consequence of new developments in experimental and theoretical methods.
